# Core Symptoms of Major Depressive Disorder among Palliative Care Patients

**DOI:** 10.3390/ijerph15081758

**Published:** 2018-08-16

**Authors:** Ng Su Huey, Ng Chong Guan, Jesjeet Singh Gill, Koh Ong Hui, Ahmad Hatim Sulaiman, Sharmilla Kanagasundram

**Affiliations:** 1 Hospital Bahagia Ulu Kinta, Jalan Besar, 31259 Tanjong Rambutan, Perak, Malaysia; suhueyng@hotmail.com; 2 Department of Psychological Medicine, Faculty of Medicine, University of Malaya, 50603 Kuala Lumpur, Malaysia; jesjeet@um.edu.my (J.S.G.); ohkoh@um.edu.my (K.O.H.); hatim@um.edu.my (A.H.S.); sharmilla_thanasan@um.edu.my (S.K.)

**Keywords:** depression, criteria, palliative, diagnosis, prevalence

## Abstract

A valid method to diagnose depression in palliative care has not been established. In this study, we aim to determine the prevalence of depression and the discriminant validity of the items of four sets of diagnostic criteria in palliative care. This is a cross-sectional study on 240 palliative care patients where the presence of depression was based on the Diagnostic and Statistical Manual of Mental Disorders, DSM–IV Criteria, Modified DSM–IV Criteria, Cavanaugh Criteria, and Endicott’s Criteria’s. Anxiety, depression, and distress were measured with Hospital Anxiety and Depression Scale and Distress Thermometer. The prevalence of depression among the palliative care patients was highest based on the Modified DSM–IV Criteria (23.3%), followed by the Endicott’s Criteria (13.8%), DSM–IV Criteria (9.2%), and Cavanaugh Criteria (5%). There were significant differences (*p* < 0.05) in the depressive symptoms showed by DSM–IV item 1 (dysphoric mood), item 2 (loss of interest or pleasure), and Endicott’s criteria item 8 (brooding, self-pity, or pessimism) among the palliative patients, even after adjustment for the anxiety symptoms and distress level. We found that dysphoric mood, loss of interest, and pessimism are the main features of depression in palliative patients. These symptoms should be given more attention in identifying depression in palliative care patients.

## 1. Background

In Malaysia, there are many patients with underlying terminal illness undergoing palliative care, which comprises of consultative palliative care services, inpatient palliative care units, outpatient clinics, and community palliative care and day care services [1]. Palliative care patients are often vulnerable to psychological distress that can range from mild feelings of sadness and vulnerability to significant depression and anxiety [2]. Psychological distress affects patients in all aspects of life such as impairing their capacity to experience pleasure and meaning [3], to interact with their loved ones, and to arrange their practical and financial affairs [4]. Their ability to participate in treatment and cope with their illness is also affected [2]. 

Major depressive disorder is a common mental health problem that causes significant mortality and morbidity. It does not only affect a person’s emotional state but also disrupts their functioning in both occupational and social aspects [5]. It is projected that by the year 2020, major depressive disorder will be the second leading cause of disability after ischemic heart disease [6]. Depression in palliative care patients is very common, and the exact underlying cause of depression among palliative care patients is most likely to be multifactorial. Depression in palliative care patients not only causes significant distress to them but also their caregivers. It reduces their quality of life [7] and is a risk factor for both suicide and desire to hasten death [8,9]. Unfortunately, depression in palliative care patients is often under-detected and under-treated [10].

It is difficult to distinguish between depression and grief or appropriate sadness in palliative care patients, as they share similar signs and symptoms [4,11] and there are no diagnostic tests, biomarkers, or physical signs to assist in making a diagnosis [10]. Up to now, the standard method to diagnose depression in palliative care patients has not been established.

There are several approaches in establishing a diagnosis of major depressive disorder. The most common standard approach is using a Structured Clinical Interview for DSM–IV (SCID) [12]. This etiological approach gives a more precise view on the presence of depression, as these interviews will only include symptoms of depression if it is not attributed to a medical or physical illness. The disadvantage of this etiologic approach is that the interviewer is unlikely to have adequate knowledge on all medical illness to be able to determine whether the symptoms arise from a medical condition or are due to depression instead [13]. There is a risk of false positives if these are used in patients with severe physical illness, as they may experience somatic or physical symptoms such as disturbed sleep, poor appetite and loss of weight, poor concentration, and fatigue as a result of their underlying terminal illness rather than the symptoms of depression [4,12].

Several different approaches have been recommended to overcome this diagnostic problem among terminally ill patients. Three types of different approaches have been employed to identify depression among cancer patients: inclusive, exclusive, and substitutive approaches [13,14]. The inclusive approach includes all depressive symptoms regardless of whether or not the symptoms may be attributable to medical illness. Modified DSM–IV Criteria uses this approach to identify depression. However, this approach has the tendency to over-diagnose patients who are medically ill with depression and causes false positive results due to its high sensitivity and lower specificity [13,14]. This was demonstrated by Kathol et al. (1990), who found that the prevalence of depression in cancer patients dropped by 8% when comparing an inclusive approach with an etiologic approach that removes depressive symptoms attributed to cancer [15].

The substitutive approach replaces the somatic or physical symptoms (disturbed sleep, poor appetite or loss of weight, poor concentration, and fatigue) of DSM–IV Criteria with non-somatic symptoms (fearfulness or depressed appearance, social withdrawal or decreased talkativeness, brooding, self-pity, or pessimism, and cannot be cheered up, does not smile, no response to good news or funny situations). Endicott proposed such an approach called Endicott’s Criteria to diagnose depression in cancer patients [13,14,15,16]. The prevalence of depression using the Endicott’s Criteria was found to be similar to the inclusive approach in another study [15]. The last approach used in assessing depression in cancer patients and those who are medically ill is the exclusive approach (Cavanaugh Criteria), whereby the somatic symptoms of depression are removed (changes in weight or appetite, sleep disturbance, and fatigue are replaced with symptoms such as not participating in medical care in spite of ability to do so, not progressing despite improving medical condition, and/or in functioning at a lower level than the medical condition warrants) from the diagnostic criteria. This approach can lead to false negatives with lower prevalence of depression due to its restricted criteria [13,14].

As studies to assess the prevalence of major depressive disorder among palliative care patients in a local setting and comparisons of the four sets of diagnostic criteria (DSM–IV Criteria, Modified DSM–IV Criteria, Cavanaugh Criteria, and Endicott’s Criteria) have not been carried out before in Malaysia, this study was carried out in two Malaysian centers, an urban and a semi-urban setting, to determine the prevalence of major depressive disorder based on the four sets of diagnostic criteria mentioned, and to determine the best diagnostic criteria to be used in palliative care patients in Malaysia.

## 2. Methods

This is a multi-center cross-sectional study to determine the prevalence of major depressive disorder among palliative care patients based on the four sets of diagnostic criteria (DSM–IV Criteria, Modified DSM–IV Criteria, Cavanaugh Criteria, and Endicott’s Criteria). The discriminant validity of each items of the four sets of diagnostic criteria was examined.

The data collection was first started at University Malaya Medical Centre (UMMC) from June 2011 to May 2012 and then followed by Hospital Raja Permaisuri Bainun (HRPB) from July to November 2012. Patients were recruited from both the palliative clinic and palliative ward at HRPB and palliative ward at UMMC.

The inclusion criteria for this study were patients aged 18 years and above who were receiving palliative care, patients who were able to communicate, and patients who were able to give consent. Those who were confused or too weak or unwell to participate in this study were excluded. 

### 2.1. Procedure

The socio-demographic characteristics of the eligible subjects (age, gender, ethnicity, religion, education level, marital status, and number of children) were collected. The information on clinical characteristic (past psychiatric history of depression, family history of depression, treatment, and medical co-morbidity) were also collected.

The presence of depressive symptoms was screened with Hospital Anxiety and Depressive Scale (HADS). Hospital Anxiety and Depression Scale (HADS) is a self-report screening instrument devised by Zigmond and Snaith to screen for both depression and anxiety. It is easy to administer and excludes physical and somatic symptoms [17]. It is also reliable and has been validated for palliative care patients [18,19]. It has 14 questions with two subscales (HADS-A: seven questions for anxiety and HADS-D: seven questions for depression). It is scored on a four-point Likert scale (0–3) to give subscale scores for both anxiety and depression ranging from 0 to 21. A score of 0–7 was categorized as having no problem, 8–10 as borderline abnormal, and 11–21 as abnormal [17]. The validated Malay version of HADS has a sensitivity of 92.3% and specificity is 90.8% when a cut-off score of 8/9 is used to detect depression [20].

Distress was measured using the Distress Thermometer, which is a self-report screening instrument measuring distress (ranges from sadness, vulnerability, and fears to significant depression, anxiety, panic, etc.) in patients with cancer. This rapid screening instrument uses a 0 to 10 visual analogue scale with no distress at 0, moderate distress at midpoint, and extreme distress at 10. It was developed by the National Comprehensive Cancer Network (NCCN) and has been validated. A score of 4 and above indicates a clinically significant distress level [2].

Patients were then interviewed using the four sets of diagnostic criteria of major depressive disorder (DSM–IV Criteria, Modified DSM–IV Criteria, Cavanaugh Criteria, and Endicott’s Criteria).

### 2.2. Statistical Analysis

Descriptive analyses were conducted for the socio-demographic characteristics of the study subjects. The mean differences of the depressive symptoms based on HADS-depressive sub-scores of each item of all the diagnostic criteria were determined. The adjusted mean differences were further examined by including HADS-anxiety sub-scores and distress scores as covariates using logistic regression analysis. All tests were two-tailed with a significant level of 0.05.

### 2.3. Ethical Considerations

Permission to carry out the study was granted by the Medical Ethics committee of UMMC (10716) and from the Director of HRPB, Ipoh (NMRR-11-1095-10716). Consent was obtained from each patient before he or she entered the study.

## 3. Results

A total of 240 eligible patients were recruited: 150 (62.5%) patients from HRPB and 90 (37.5%) patients from UMMC. The mean age of the patients was 62.13 years (SD = 12.830) with mainly female patients (57.1%). The largest ethnic group represented were Chinese (*n* = 153, 63.8%), and most were Buddhist (*n* = 124, 51.7%). As for education, only 13.3% (*n* = 32) of the patients had no formal education. Most of the subjects were married at the time of the study (*n* = 157, 65.4%), and 57.1% (*n* = 137) of them had more than two children (Table 1).

Table 2 shows the frequency of each item being reported among the four different diagnostic criteria. The first five items in DSM–IV Criteria are similar to the Modified DSM–IV Criteria and Endicott’s Criteria, while the first six items in DSM–IV Criteria are similar to Cavanaugh Criteria. A high number of patients reported the presence of symptoms from Modified DSM–IV Criteria item 7 (*n* = 133, 55.4%), item 8 (*n* = 130, 54.2%), and item 9 (*n* = 155, 64.6%). There was also a high rate of reporting for DSM–IV Criteria item 1 (*n* = 62, 25.8%), item 2 (*n* = 66, 27.5%), and item 3 (*n* = 90, 37.5%). Prevalence of major depressive disorder was the highest when the Modified DSM–IV Criteria was used (*n* = 56, 23.3%), followed by the Endicott’s Criteria (*n* = 33, 13.8%) and the DSM–IV Criteria (*n* = 22, 9.2%), and lowest for the Cavanaugh Criteria (*n* = 12, 5%).

Table 3 showed the discriminant validity of depression in the items. Item 1 (dysphoric mood) and item 2 (loss of interest or pleasure) from DSM–IV and item 8 (brooding, self-pity, or pessimism) from Endicott are the only diagnostic criteria that significantly differentiate the level of depression among the study subjects after having adjusted for the underlying anxiety and distress level.

## 4. Discussion

One of the objectives of this study was to determine the prevalence rates of major depressive disorder among palliative care patients based on the four different sets of diagnostic criteria (DSM–IV Criteria, Modified DSM–IV Criteria, Cavanaugh Criteria, and Endicott’s Criteria) and to propose which could be the most suitable diagnostic criteria to diagnose major depressive disorder in this group of patients. The highest rates were found using the Modified DSM–IV Criteria, followed by the Endicott’s Criteria and the DSM–IV Criteria, and the lowest using the Cavanaugh Criteria. These findings were expected, as Modified DSM–IV Criteria includes somatic symptoms, which are common among palliative care patients.

However, Kathol et al. (1990) found the prevalence of depression using the Endicott’s Criteria, a substitutive approach, to be similar to the use of an inclusive approach [15]. This differs from our study, where the prevalence of depression using the Endicott’s Criteria was lower than the inclusive Modified DSM–IV Criteria approach but higher than DSM–IV Criteria, which utilizes an etiological approach. These differences may be due to the different characteristics of patients being studied. In this study, the samples consisted of both cancer and non-cancer palliative patients while samples in the study by Kathol et al. consisted of patients who were suffering from terminal solid tumors. The study by Kathol et al. also had a smaller sample size compared to this study [15,17].

Among all the items, item 1 (dysphoric mood) and item 2 (loss of interest or pleasure) from DSM–IV and item 8 (brooding, self-pity, or pessimism) from Endicott were the most common symptoms reported among the depressive patients. This is not surprising, as many patients with terminal illness often have somatic complaints such as pain and fatigue that leads to dysphoric mood, loss of interest or pleasure, and pessimism.

As in our study, dysphoria has been found to be the most common symptom in any major depressive disorder regardless of the type of population studied [21]. Dysphoric mood will ultimately lead to feelings of hopelessness, worthlessness, or helplessness [22,23], and ultimately individuals to isolate themselves from others [24].

Loss of interest or pleasure, one of the most common symptoms found, is another obvious symptom that can be found among the depressed patients of any population. Research has found that loss of interest or pleasure are considered to be essential requirements in diagnosing a depressive episode [25,26]. Loss of interest or pleasure significantly interferes in one’s sexual functioning [27], which tends not only to distress the individual, but also tends to strain relationships, which may worsen the depression, hence creating a vicious cycle [27]. 

With negative thoughts, an individual will automatically become pessimistic, with self-pity and brooding setting in [28]. It is a feeling of unhappiness that is able to alter one’s perception of reality. Brooding, self-pity, and pessimism are viewed as classical depressive personality traits [29]. Hence, it is not surprising that this group of “traits” represented some of the most common symptoms found in our study.

Our findings are consistent with a study by Akechi et al. (2009) [14] that demonstrated that the somatic symptoms of DSM–IV, such as insomnia or hypersomnia, weight loss or gain or a decrease in appetite, fatigue or loss of energy, and diminished ability to think or concentrate, were not useful for making a diagnosis of depression or assessing its severity among patients with cancer. He has also demonstrated that suicidal ideation will only be reported when the severity of depression reaches a moderate level. This is in keeping to what we found where item 5 of the DSM–IV Criteria (suicidal ideation) was not significantly associated with any variables and therefore not useful in detecting depression in palliative care patients.

In general, depressed mood, loss of interest and brooding, and self-pity or pessimism were observed to have played a strong role among the depressed patients. Based on the results and past findings, it is obvious to see that these three symptoms are the key symptoms.

The findings of this study have to be interpreted with caution due to several limitations that arose mainly from the study design and methods. The limitations may limit the extent to which we can generalize the findings of the study. Therefore, the limitations of this study should be taken into consideration when evaluating the results of the study as a whole.

First, the majority of the patients in this study were of Chinese ethnicity, which does not reflect the true distribution of ethnic groups in Malaysia. The disproportionate ethnic distribution in this study may be partly due to the location where the study was carried out. The ethnic distribution of the patients in the study could affect the results of this study and, as such, we may not be able to generalize the findings to other palliative populations.

Second, there were some potential limitations surrounding the validity of the language of the questionnaires used in this study, as, at times, some patients required assistance due to their limited command of English or Malay. In order to minimize this error, these patients were assisted by the rater using a Malay version of the questionnaire. Third, the sampling method in this study was based on convenient sampling instead of random sampling due to limited time and resources and this may affect the generalizability of the results. This study was not able to capture a subgroup of terminally ill patients who refused treatment or refused to attend a palliative clinic and this subgroup of patients may be suffering from depression. Fourth, information was obtained from patients and this can lead to recall bias. Information regarding patient characteristics can be verified with family members accompanying a patient in order to improve the accuracy of the information. The presence or absence of depressive symptoms according to items in DSM–IV Diagnostic Criteria, Modified DSM–IV Criteria, Cavanaugh Criteria, and Endicott Criteria were obtained through interviews conducted by investigators and this could have been a source of response bias. However, this was minimized by using open-ended questions. Fifth, this was a cross-sectional study. Therefore, we were only able to measure the association between factors and were unable to establish cause and effect or direction of relationships. Sixth, the study was using DSM–IV diagnostic criteria for the etiological and inclusive approach while assessing the patients. We believed that the application of old diagnostic criteria will not affect the validity of the current result, as there were not many changes in DSM–5 diagnostic criteria for major depressive disorder. Lastly, we grouped and analyzed all the patients together, regardless of the underlying medical conditions. However, the objective of the current study intended to identify the core symptoms of depression of palliative care patients in general.

## 5. Conclusions

Though this study, we were able to determine which items in DSM–IV Criteria, Modified DSM–IV Criteria, Endicott’s Criteria, and Cavanaugh Criteria have the ability to differentiate between depressed and non-depressed palliative care patients. We propose that these items (dysphoric mood; loss of interest or pleasure; and brooding, self-pity, or pessimism) should be given more attention in identifying palliative care patients who are suffering from major depressive disorder.

## Figures and Tables

**Table 1:** Socio-demographic characteristics of palliative care patients (*n* = 240).

	Mean (SD)	N (%)
**Centre** Hospital Raja Permaisuri Bainun University Malaya Medical Centre		150 (62.5) 90 (37.5)
**Age**	62.13 (12.830)	
**Gender** Male Female		103 (42.9) 137 (57.1)
**Ethnicity** Malay Chinese Indian Others		58 (24.2) 153 (63.8) 23 (9.6) 6 (2.5)
**Religion** Muslim Christian Buddhist Hindu Others		61 (25.4) 35 (14.6) 124 (51.7) 16 (6.7) 4 (1.7)
**Education Level** Primary Secondary Tertiary None		91 (37.9) 96 (40.0) 18 (7.5) 32 (13.3)
**Marital Status** Single Married Widowed Divorced/Separated		36 (15.0) 157 (65.4) 39 (16.3) 8 (3.3)
**Number of children** ≤2 >2		103 (42.9) 137 (57.1)

Percentages may not add up to 100% due to rounding.

**Table 2:** Frequency of each item in the four different diagnostic criteria.

	N (%)
DSM–IV Item 1 (dysphoric mood) DSM–IV Item 2 (loss of interest/pleasure) DSM–IV Item 3 (psychomotor agitation/retardation) DSM–IV Item 4 (feelings of worthlessness/self-reproach/guilt) DSM–IV Item 5 (thoughts of death/suicidal ideation or attempt) DSM–IV Item 6 (diminished ability to think/concentrate/indecisiveness) DSM–IV Item 7 (weight loss/gain or decrease in appetite) DSM–IV Item 8 (insomnia/hypersomnia) DSM–IV Item 9 (fatigue/loss of energy)	62 (25.8) 66 (27.5) 90 (37.5) 45 (18.8) 14 (5.8) 12 (5.0) 23 (9.6) 25 (10.4) 22 (9.2)
Modified DSM–IV Item 6 (diminished ability to think/concentrate) Modified DSM–IV Item 7 (weight loss/gain or decrease in appetite) Modified DSM–IV Item 8 (insomnia/hypersomnia) Modified DSM–IV Item 9 (fatigue/loss of energy)	27 (11.3) 133 (55.4) 130 (54.2) 155 (64.6)
Cavanaugh Criteria Item 7 (not participating in medical care in spite of ability to do so/not progressing despite improving medical condition/functioning at a lower level than the medical condition warrants)	6 (2.5)
Endicott Criteria Item 6 (fearfulness/depressed appearance) Endicott Criteria Item 7 (social withdrawn/decreased talkativeness) Endicott Criteria Item 8 (brooding/self-pity or pessimism) Endicott Criteria Item 9 (cannot be cheered up/does not smile/no response to good news or funny situations)	44 (18.3) 35 (14.6) 42 (17.5) 11 (4.6)

**Table 3:** Discriminant of depressive symptoms based on HADS-depressive subscale for each item.

	Depressive Symptoms Based on HADS-Depressive Subscale			
	Means	Adjusted Odds *	95% CI	*p* Value
DSM–IV Item 1 Yes No	12.29 (3.78) 6.75 (3.88)	0.92	0.86, 0.99	0.03
DSM–IV Item 2 Yes No	12.35 (3.60) 6.63 (3.83)	0.88	0.82, 0.94	<0.01
DSM–IV Item 3 Yes No	12.15 (4.02) 7.69 (4.37)	0.97	0.89, 1.04	0.38
DSM–IV Item 4 Yes No	12.13 (3.96) 7.28 (4.18)	1.01	0.94, 1.10	0.751
DSM–IV Item 5 YesNo	13.57 (3.76) 7.86 (4.49)	0.91	0.81, 1.03	0.14
DSM–IV Item 6 Yes No	14.50 (3.85) 7.86 (4.34)	0.95	0.84, 1.08	0.41
DSM–IV Item 7 Yes No	12.26 (4.64) 7.76 (4.33)	0.97	0.89, 1.05	0.44
DSM–IV Item 8 Yes No	12.04 (3.92) 7.75 (4.41)	1.00	0.92, 1.08	1.00
DSM–IV Item 9 Yes No	12.77 (4.13) 7.73 (4.34)	0.96	0.88, 1.04	0.30
Modified DSM–IV Item 6 Yes No	12.26 (4.49) 7.68 (4.30)	1.01	0.93, 1.10	0.78
Modified DSM–IV Item 7 Yes No	9.29 (4.55) 6.83 (4.18)	0.97	0.92, 1.03	0.33
Modified DSM–IV Item 8 Yes No	9.45 (4.44) 6.70 (4.25)	1.01	0.96, 1.07	0.69
Modified DSM–IV Item 9 Yes No	9.15 (4.25) 6.46 (4.60)	0.98	0.91, 1.04	0.46
Cavanaugh Criteria Item 7 Yes No	12.20 (4.18) 7.28 (4.13)	0.94	0.88, 1.01	0.11
Endicott Criteria Item 6 Yes No	12.74 (3.76) 7.41 (4.21)	0.95	0.88, 1.03	0.18
Endicott Criteria Item 7 Yes No	11.93 (4.03) 7.39 (4.26)	1.01	0.94, 1.09	0.73
Endicott Criteria Item 8 Yes No	15.09 (3.86) 7.86 (4.32)	0.79	0.67, 0.93	0.01
Endicott Criteria Item 9 Yes No	8.00 (3.69) 8.2 (4.58)	1.01	0.83, 1.23	0.90

* Adjusted for the underlying anxiety subscale scores and distress scores based on logistic regression analysis. HADS: Hospital Anxiety and Depression Scale; DSM: Diagnostic and Statistical Manual of Mental Disorders.
